# Olfactory Imprinting of Amino Acids in Lacustrine Sockeye Salmon

**DOI:** 10.1371/journal.pone.0008633

**Published:** 2010-01-08

**Authors:** Yuzo Yamamoto, Hiroshi Hino, Hiroshi Ueda

**Affiliations:** Laboratory of Aquatic Bioresources and Ecosystem, Field Science Center for Northern Biosphere and Division of Biosphere Science, Graduate School of Environmental Science, Hokkaido University, Sapporo, Hokkaido, Japan; Freie Universitaet Berlin, Germany

## Abstract

Juvenile salmon have an olfactory ability to imprint their natal stream odors, but neither the odor properties of natal stream water nor the imprinting timing and duration have been clarified as yet. Here we show, using electrophysiological and behavioral experiments, that one-year-old lacustrine sockeye salmon (*Oncorhynchus nerka*) can be imprinted around the stage of parr-smolt transformation (PST) by a single amino acid, 1 µM L-proline (Pro), or L-glutamic acid (Glu). We also show by real-time PCR that changes occur in mRNA levels of the salmon olfactory imprinting-related gene (SOIG) around PST. The electro-olfactogram (EOG) responses of test fish exposed to Pro in March (before PST) and April–June (during PST) for 2 weeks were significantly (1.7-fold) greater than those of non-exposed control fish, but not those of test fish exposed in July (after PST). When Pro and control water were added to the water inlets of a two-choice test tank during the spawning season 2 years after the test water exposure, 80% of maturing and matured test fish exposed before and during PST showed a preference for Pro, whereas those exposed after PST did not. The EOG response of test fish exposed to Pro or Glu for 1 hour, 6 hours, 1 day, 7 days, or 14 days in May revealed that only the response after 14 days of exposure was significantly (1.8-fold) greater than the control. The expression levels of SOIG mRNA increased before and during PST, and decreased after PST. We conclude that one-year-old lacustrine sockeye salmon can be imprinted by a single amino acid before and during PST, and that imprinting requires exposure for at least 14 days.

## Introduction

Adult salmon are well known for their accurate homing, guided by an olfactory memory of their natal stream that is imprinted during their juvenile stage [1]. Two different olfactory hypotheses have been proposed for salmon imprinting and homing: one is the imprinting hypothesis based on coho salmon (*Oncorhynchus kisutch*) [2]; the other is the pheromone hypothesis based on Arctic char (*Salvelinus alpines*) and Atlantic salmon (*Salmo salar*) [3], [4]. The pheromone hypothesis assumes that juvenile salmon in a stream release population-specific odors that guide homing adults. However, there are no juveniles of chum salmon (*O. keta*) or pink salmon (*O. gorbuscha*) present at the time that the adults return. It is now widely accepted that some specific odorant factors in the natal stream are imprinted on the olfactory system of juvenile salmon during downstream migration, and that adult salmon use these factors to recognize their natal stream during homing migration [5]–[7]. However, neither the odor properties of natal stream water nor the imprinting mechanisms, timing and duration have been clarified as yet.

Studies on the formation of memory have recently concentrated on the possible role of long-term potentiation (LTP) in learning and memory, with a focus on the *N*-methyl-_D_-aspartate (NMDA) receptor, which induces LTP. LTP is known to occur in the brain of zebrafish (*Danio rerio*) [8], rainbow trout (*O. mykiss*) [9], and common carp (*Cyprinus carpio*) [10]. In addition, the gene coding the NMDA receptor has also been reported to be expressed in the brain of rainbow trout [11].

Previous imprinting experiments were mainly conducted using juvenile coho salmon that had been imprinted with either β-phenylethyl alcohol (PEA) or morpholine during parr–smolt transformation (PST), and that were lured into unfamiliar streams scented with these odorants during homing migration a few years later [12], [13]. The olfactory receptor cells of coho salmon that had been imprinted with PEA had a higher sensitivity to PEA as compared with non-imprinted fish [14]. In case of juvenile coho salmon that had been exposed to PEA or natural stream odorants at various stages of development, only fish that were exposed to these odorants during PST formed an imprinted memory [15].

Imprinting in the olfactory system is now thought to be due to sensitization of the peripheral sensory neurons to specific odorants. Guanylyl cyclase is thought to play a modulatory role in intracellular signaling in vertebrate olfactory receptor cells. Dittman et al [16] examined the sensitivity of olfactory adenylyl and guanylyl cyclases to PEA during different developmental stages in coho salmon. Their results showed that exposing salmon to PEA during PST resulted in a sensitization of olfactory cilia guanylyl cyclase to PEA. Changes in guanylyl cyclase were observed only during the brief period prior to spawning when an increase in olfactory sensitization is crucial for natal stream recognition in the wild.

The chemical properties of natal stream odors have been examined mainly by electrophysiological studies, which suggested that the stimulatory portion of the natal stream water was non-volatile [17]. Natal stream water odorants have been reported to be absorbed on activated carbon and ion-exchange resin, insoluble in petroleum ether, dialyzable, non-volatile, and heat-stable from spectral analysis of the olfactory bulbar response [18]. On the basis of our recent electrophysiological experiments, we proposed that amino acids dissolved in the natal stream water might be odorant substances for masu salmon (*O. masou*) [19]. Behavioral experiments further demonstrated that mature chum salmon were attracted to an artificial solution consisting of the same amino acid composition as their natal stream water [20], [21]. These results from electrophysiological and behavioral experiments suggest the possibility that amino acids dissolved in natal stream water are possible natal stream odors for salmon. Morpholine, which has been used in previous imprinting experiments, is an artificial substance and is not found in natural stream water, whereas amino acids are present in natural stream water.

Recently, we identified the salmon olfactory imprinting-related gene (SOIG) in the olfactory system of one-year-old lacustrine sockeye salmon by using the subtractive hybridization technique of representational difference analysis (cDNA-RDA) [22].The predicted open reading frame (756 bp) of SOIG encodes a protein of 252 amino acids and shares low amino acid sequence identity with the urokinase-type plasminogen activator receptor (u-PAR) [23]. u-PAR belongs to a member of the Ly-6 superfamily that is found in several species [24]–[26]. A Ly-6-related protein (*odr*-2) has been isolated from *Caenorhabditis elegans*, and it has been suggested that *odr-2* may regulate olfactory neuron signaling within the neuronal network required for chemotaxis [27]. Although the precise function of SOIG has not been clarified as yet, SOIG may have important roles in olfactory imprinting in lacustrine sockeye salmon. Thus, it would be interesting to examine changes in the expression levels of SOIG mRNA around PST by a real-time polymerase chain reaction (PCR) technique.

Juvenile lacustrine sockeye salmon (*O. nerka*) in Lake Toya and Lake Shikotsu, Hokkaido, Japan, are spawned and released from hatcheries within a few months of emergence, and adults attain maturity in 3–5 years and return to their natal hatcheries for breeding. The active spawning season is the middle of October to early November, but maturing adults gather near the shore of the hatcheries as early as September. The average homing percentage of lacustrine sockeye salmon in Lake Shikotsu was found to be 83% in both sexes, but these percentages varied depending on gonadal maturity [28]. The smolting process in lacustrine sockeye salmon is not well understood; however we have found that the body color of one-year-old fish is silver and their fins are clear with intense black pigment in May, the condition factor (CF) is significantly low in May and June (Figure S1), and serum thyroxine (T_4_) levels peak in May (Figure S2). From these data, we designated the period from April to June as PST in the present study.

In the present study, electrophysiological and behavioral experiments were conducted to examine whether one-year-old lacustrine sockeye salmon could be imprinted by an amino acid, L-proline (Pro) or L-glutamic acid (Glu) (Table S1) before (March), during (April-June), and after (July) PST. To determine whether the test fish were imprinted by a single amino acid, the electro-olfactogram (EOG) response to the test water was measured and the relative magnitude of the response was compared between exposed test fish and non-exposed control fish in June and October for three successive years. Behavioral experiments in a two-choice test tank (Y maze) were also carried out on maturing and matured test fish that had been exposed to Pro from March to July two years previously to determine whether mature fish could select Pro or not. In addition, the time required for imprinting by one amino acid, either Pro or Glu, was examined by the EOG response for 1 hour, 6 hours, 1 day, 7 days, and 14 days. Lastly, a molecular biological experiment was carried out to measure changes in SOIG mRNA expression levels by real-time PCR. These physiological, behavioral and molecular experiments are discussed in relation to the odor properties of the natal stream, along with the imprinting timing and duration of lacustrine sockeye salmon.

## Results

**Figure 1:**
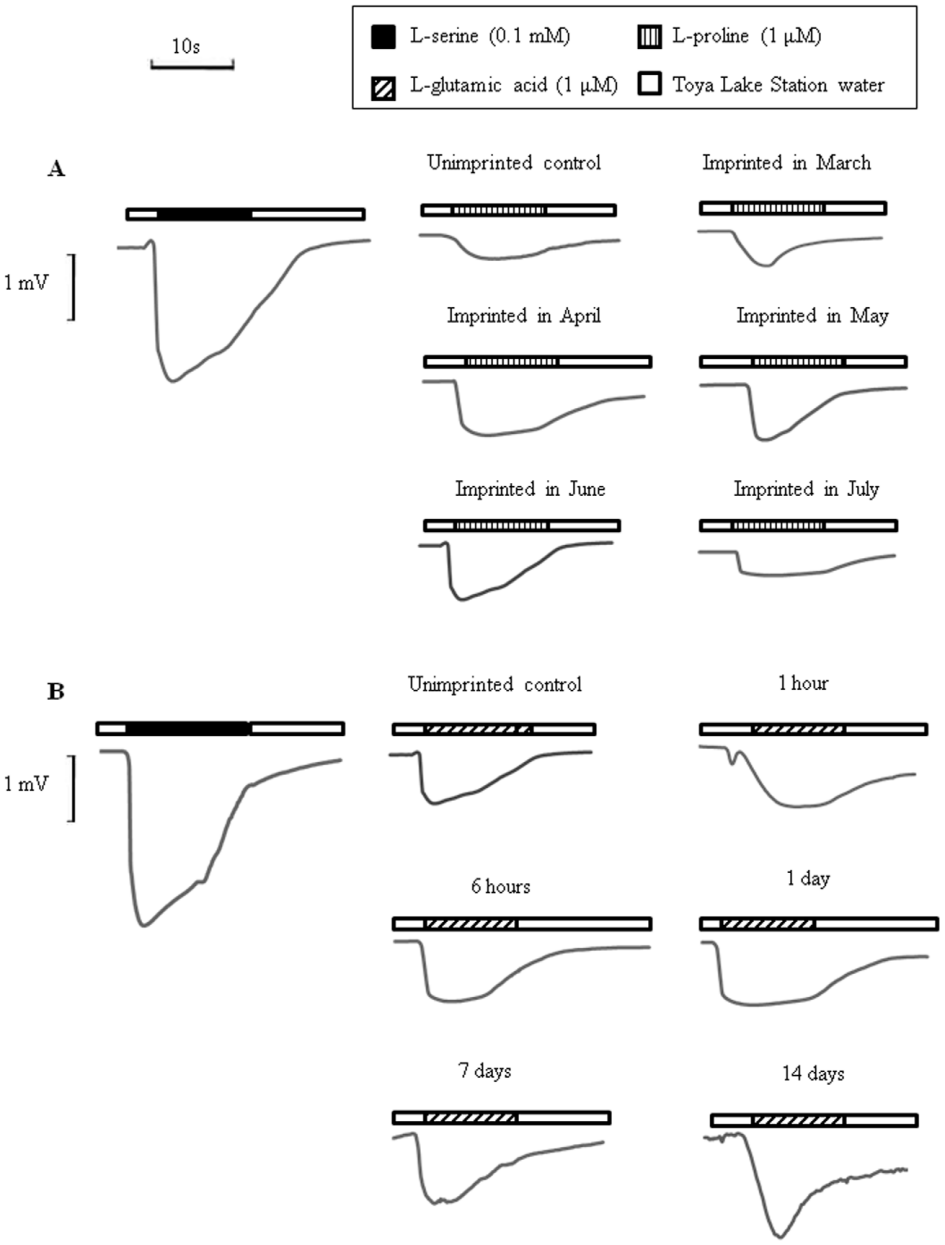
Typical electro-olfactogram (EOG) response of sockeye salmon to 0.1 mM L-serine and the test water in October of 2007. (A) unimprinted control and experimental fish exposed to 1 µM L-proline water from March to July of 2005 in the monthly variation experiment. (B) unimprinted control and experimental fish exposed to 1 µM L-glutamic acid from 1 hour to 14 days in May of 2006 in the requirement time experiment.

**Figure 2:**
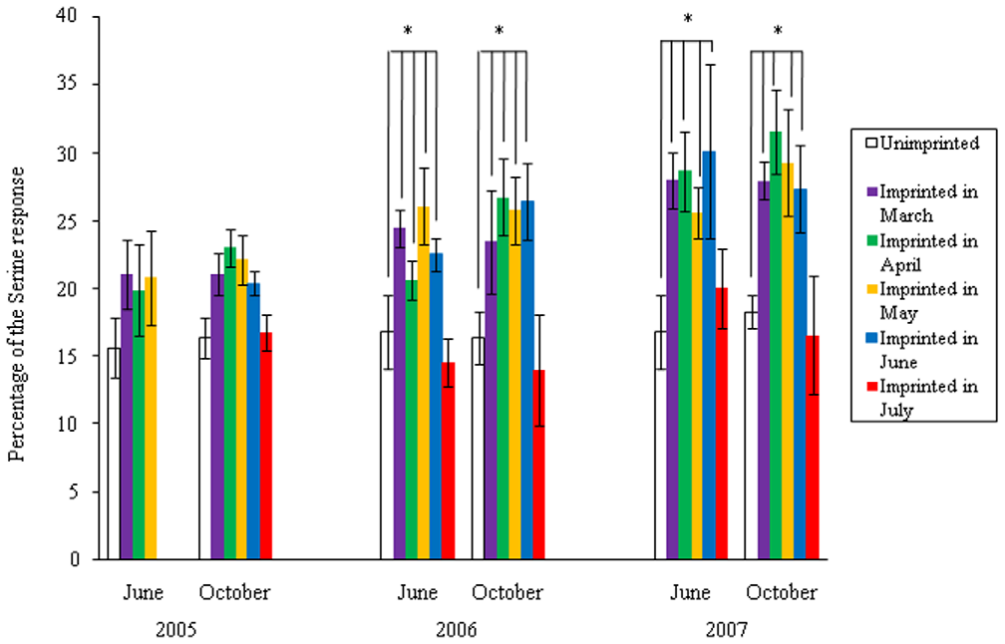
Difference in olfactory response to the test water (1 µM L-proline (Pro)) of experimental fish exposed to Pro from March to July in 2005 and unimprinted control fish. The response is expressed as a percentage of the response to 0.1 mM L-serine dissolved in distilled water. The values are means ± SEM of data obtained from six fish in each group. Significant differences between experimental fish exposed to Pro in March, April, May, and June of 2006 and 2007, and control fish are indicated (*p<0.05 by one way ANOVA followed by Dunnett's test).

The EOG response to 0.1 mM L-serine (Ser) and the test water (1 µM L-proline: Pro) of unimprinted control and experimental fish of both sexes exposed to the test water from March to July of 2005 was measured in October of 2007 (Fig. 1A). There were no differences in the EOG response between males and females. In June and October of 2005, 2006 and 2007, the relative magnitude of the EOG response of fish to the test water in the experimental and unimprinted control groups was examined as a percentage of the EOG response to 0.1 mM Ser. Although the EOG response to 1 µM Pro was between 15 and 32% of that to 0.1 mM Ser, fish exposed to the test water showed a 1.7-fold greater response than unimprinted control fish (Fig. 2). The EOG response in the fish exposed to the test water before and during PST showed a slight but not significant change in June and October of 2005, but showed significant and greater increases in June and October of 2006 and 2007 (p<0.05) as compared with the control fish. However, in fish exposed to the test water after PST, there was no significant difference between the experimental and control groups.

**Figure 3:**
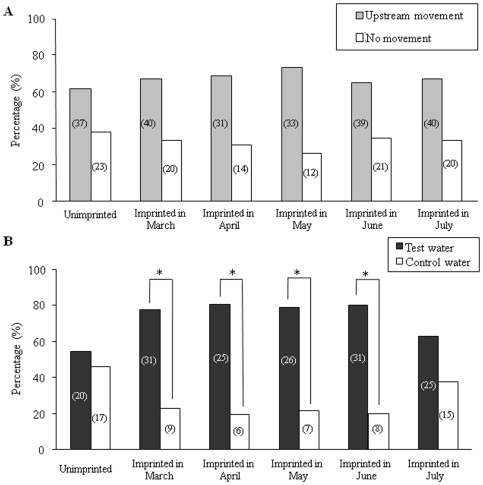
Selectivity to the test water of mature sockeye salmon in a two-choice test tank. Shown are the upstream movement (A) and selectivity (B) of mature sockeye salmon of experimental fish (exposed to 1 µM L-proline from March to July in 2005) and unimprinted control fish in a two-choice tank containing either the test water (Pro) or control water (Toya Lake water). Significant differences between the test water and control water are indicated in the groups that were exposed to Pro in March, April, May and June (*p<0.05 by chi-square test). The number in parentheses indicates the number of fish that moved to each arm.

In order to avoid courtship behavior, the behavioral experiment was carried out with only male fish. When control water (Toya Lake water) flowed from both arms, 23–32 fish (65–75% of a total of 30–45 fish in each experiment) showed upstream movement to either arm, and there was no selection for either arm (data not shown). The upstream movement and arm selection of fish in the experimental and control groups was then observed when test water and control water flowed from different arms (Fig. 3). In the monthly variation experiment from March to July, 31–40 fish showed upstream movement. Among these fish, 80% of experimental fish exposed to the test water before and during PST showed significant selection for the test water (p<0.05). In contrast, there was no selection for the test water in either experimental fish exposed after PST or control fish.

**Figure 4:**
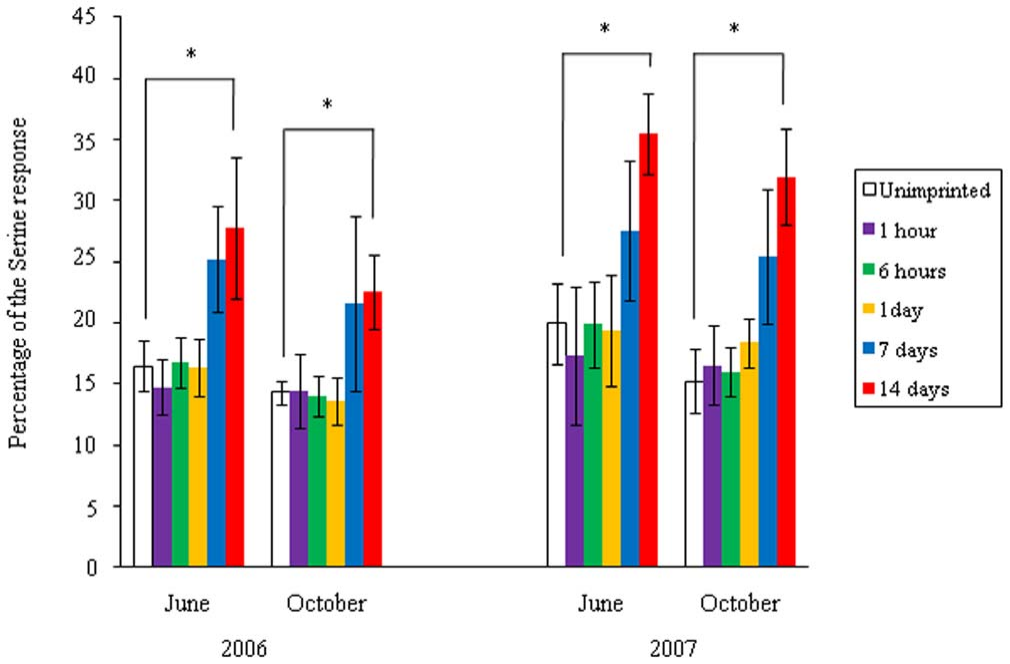
Difference in olfactory response to the test water (1 µM L-proline (Pro)) of experimental fish exposed to Pro for 1 hour, 6 hours, 1day, 7 days, and 14 days and unimprinted control fish. The response is expressed as a percentage of the response to 0.1 mM L-serine dissolved in distilled water. The values are means ± SEM of data obtained from six fish in each groups. Significant differences between the fish that were exposed to Pro for 14 days and the control group are indicated (*p<0.05 by one way ANOVA followed by Dunnett's test).

The relative magnitude of the EOG response of fish to the Pro test water was compared for experimental fish subjected to different exposure times and unimprinted control fish (Fig. 4). The responses of experimental fish exposed to the test water for 14 days were significantly greater than those of control fish (p<0.05). No significant differences were found between experimental fish exposed to the test water for 1 hour, 6 hours or 1 day, and the control group. In experimental fish exposed to the test water for 7 days, the EOG responses were different from those of the control fish, but the difference was not significant.

**Figure 5:**
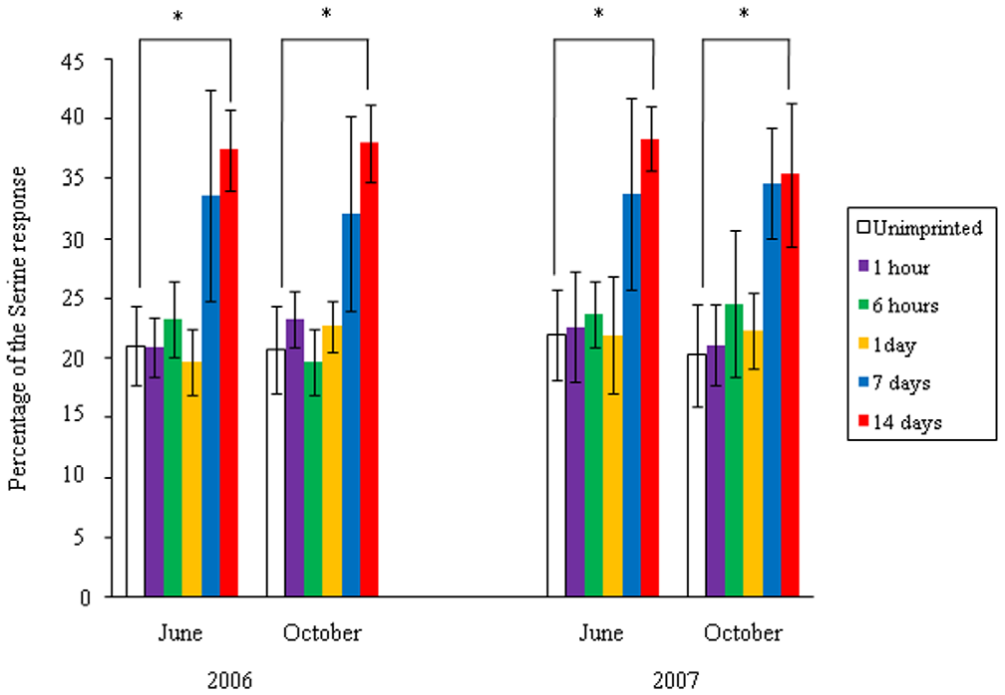
Difference in olfactory response to the test water (1 µM L-glutamic acid (Glu)) of experimental fish exposed to Glu for 1 hour, 6 hours, 1day, 7 days, and 14 days and unimprinted control fish. The response is expressed as a percentage of the response to 0.1 mM L-serine dissolved in distilled water. The values are means ± SEM of data obtained from six fish in each group. Significant differences between the fish that was exposed to Glu for 14 days and the control group are indicated (*p<0.05 by one way ANOVA followed by Dunnett's test).

The EOG to 0.1 mM Ser and the test water (1 µM L-glutamic acid: Glu) of unimprinted control and experimental fish exposed to the test water from 1 hour to 14 days in May of 2006 was measured in October of 2007 (Fig. 1B). The relative magnitude of the EOG response to the Glu test water in the experimental and control groups showed that, similar to the results of the Pro test, the responses of experimental fish exposed to the test water for 14 days were significantly greater than those of the control fish (p<0.05), whereas the responses of test fish exposed for 1 hour, 6 hours, 1 day, or 7 days were not (Fig. 5).

**Figure 6:**
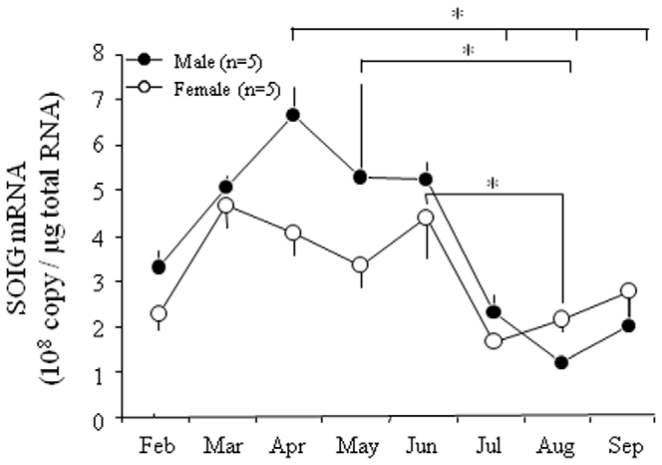
Changes in salmon olfactory imprinting-related gene (SOIG) mRNA expression levels of one-year-old lacustrine sockeye salmon from February to September of 2006. Data are represented as means±SEM. Significant differences among the months are indicated (*p<0.05 by one-way ANOVA following Tukey's test).

There were no seasonal changes in the expression level of a control housekeeping gene (β-actin). The basal levels of SOIG mRNA were 1.98−3.83×10^8^ copies/µg total RNA in both sexes of one-year-old lacustrine sockeye salmon in February. The expression levels of SOIG mRNA in males and females increased from March to June, and decreased in July (Fig. 6). There were some differences in temporal expression between males and female. In males, SOIG mRNA levels peaked in May, and were significantly higher than those from July to September. By contrast, SOIG mRNA levels in females peaked in March, then tended to decrease in April and May, and increased again in June. In particular, SOIG mRNA levels in females in June were significantly higher than those in July and August.

## Discussion

The results of our electrophysiological, behavioral and molecular biological experiments provide new information that increases our understanding of salmon imprinting by amino acids around PST. The EOG results reveal clearly that one-year-old lacustrine sockeye salmon can be imprinted by 1 µM Pro or Glu before and during PST. The behavioral results also show that maturing and matured fish that were exposed to the test water before and during PST 2 years previously have the ability to select the test water. Eighty percent of test fish imprinted before and during PST showed a preference for the test water, a proportion that is similar to the average homing percentage of lacustrine sockeye salmon in Lake Shikotsu [28]. The electrophysiological and behavioral results revealed that there were significant differences between unimprinted control fish and experimental fish imprinted by a single amino acid before and during PST, and that the timeframe for imprinting timing ended after PST.

The composition of amino acids in the different streams feeding Lake Toya varies greatly, and the electrophysiological olfactory nerve response of masu salmon to artificial stream water reconstituted on the basis of amino acid composition has been shown to closely resemble the response to the actual stream water [19]. In addition, artificial natal stream water reconstituted according to amino acid composition has attractive effects on the upstream selective movement of matured chum salmon [20], [21]. Using a patch clamp technique, olfactory receptor cells of coho salmon that had been exposed to the artificial odorant PEA at the smolt stage responded to a lower PEA concentration as compared with control PEA-naïve fish [14]. The sensitivity of olfactory guanylyl cyclase has been proposed to play an important role in olfactory imprinting in coho salmon [16].

In the EOG experiment, over the 3 successive years the relative response to the test water in June and October was higher in the last 2 years in experimental fish exposed to the test water before and during PST than in control fish. These results suggest that the olfactory receptor cells exposed to the test water can respond during not only their breeding period but also their non-breeding period. This phenomenon may be related to the life history of anadromous sockeye salmon that come back to the natal stream 4 to 5 months before the breeding season. In contrast, the olfactory receptor cells of experimental fish exposed to the test water in July were unable to respond to the test water, suggesting that the olfactory imprinting ability expires after PST.

The requirement time experiment for olfactory imprinting clearly demonstrates that exposure to the test water for 14 days is enough to create an olfactory memory of the test water in the experimental fish in May. Although exposure for 7 days did not lead to a significant difference as compared with the control fish, the relative response to the test water was altered. An artificial imprinting experiment in smolt-stage coho salmon that were exposed to PEA for 10 days demonstrated the formation of an olfactory memory of PEA in adults [14], [15]. It is reasonable to conclude that the requirement time for olfactory imprinting is about 10–14 days in sockeye and coho salmon during PST.

Our real-time PCR analysis revealed that the expression levels of SOIG mRNA in the olfactory epithelium of lacustrine sockeye salmon increase from March to June, a time span that includes the period before and during PST. We observed that SOIG mRNA was expressed in both the olfactory receptor cells and basal cells of the olfactory epithelium by *in situ* hybridization, which suggested that SOIG expression might be related to cell proliferation during PST [22]. Olfactory receptor cell proliferation was reported to be induced by thyroid hormone in coho salmon [29]. There might be an important correlation between changes in SOIG m RNA expression levels and serum T_4_ levels in lacustrine sockeye salmon during PST. Expression changes in the odorant receptor (OR) gene during PST have been measured in Atlantic salmon, demonstrating that transient increases in OR transcripts are coincident with PST [30]. We recently cloned one OR gene (LSSOR1) from lacustrine sockeye salmon and additionally characterized four Pacific salmon (pink, chum, masu salmon and rainbow trout) clones with high sequence homology (96–99%) to each other [31]. However, the odorant ligands that bind these ORs have not been characterized as yet. Further intensive molecular biological studies will enhance our understanding of the cellular mechanisms of olfactory imprinting and homing in salmon.

Many physiological changes occur during PST, such as surges in plasma levels of T_4_ [32]–[34] that might be involved in olfactory imprinting [1]. Using *in vitro* autoradiography, the olfactory epithelium of smolting masu salmon was found to be enriched in thyroid hormone receptors as compared with that of parr [35]. Electrophoretic changes in olfactory system proteins was investigated in masu salmon during PST, and demonstrated that several protein spots appeared and disappeared during the course of smolting [36]. The olfactory nerve and glomerular structures in the olfactory bulb grow dramatically during PST in chinook salmon (*O. tshawytscha*) [37], and chemical and structural changes in the brain have been examined during PST in coho salmon [38], [39]. PST was reported to be the critical period for olfactory imprinting in coho salmon exposed to PEA as embryo, parr, and smolt, whereby only salmon exposed to PEA at the smolt stage showed increased attraction to PEA as adults [15]. However, Tilson et al [40], [41] showed that kokanee salmon (similar to lacustrine sockeye salmon) displayed olfactory imprinting of artificial odorants as alevins and emergent fry, as well as at the smolt stage. The timing of the commencement of olfactory imprinting in juvenile salmon before PST should be examined from the alevin stage.

In conclusion, the present study shows that one-year-old lacustrine sockeye salmon can be imprinted by a single amino acid. The olfactory imprinting occurs before and during PST, but not after PST. The odorant memories of one amino acid are maintained not only in the spawning season but also in the non-spawning season. The requirement time for imprinting is likely to be at least 2 weeks during the month of May. Further molecular and sensory biological approaches, which are currently in progress in our laboratory, will clarify the neurobiological mechanisms of olfactory imprinting and homing in salmon.

## Materials and Methods

### Experimental Animals

One-year-old lacustrine sockeye salmon [average fork length (FL), 9.6±0.8 cm; average body weight (BW), 6.9±0.9 g] hatched in 2004 and 2005 at Toya Lake Station, Field Science Center for Northern Biosphere, Hokkaido University, were used in the present study. They were reared in 1400-L circular tanks under a natural photoperiod with a constant flow of spring water at 9.8–10.7°C, and raised on standard commercial salmon pellets. The gonadal status of fish in the monthly variation experiment was immature in 2006, and pre-mature and mature in June and October 2007, respectively. The gonadal status of fish in the requirement time experiment was immature in 2006 and 2007. The condition factor (CF) was calculated by [BW (g)/FL (cm)^3^]×10^3^ (Figure S1).

Serum samples were collected from the caudal vasculature of 5 males and females. Serum levels of thyroxine (T_4_) were measured by time-resolved fluoroimmunoassay (TR-FIA) method using the protocols for T_4_ assays developed by Yamada et al [42] (Figure S2). Antiserum for T_4_ was immobilized at the surface of the microtiter plates (Wallac Oy, Turku, Finland) by physical adsorption for 18 hr at 4°C. After three washes with 0.9% saline, the plates were blocked with a blocking solution (50 mM Na_2_HPO_4_, 3% sucrose, 0.1% bovine serum albumin (BSA), 0.05% NaN_3_) for 1 hr at room temperature. The plate was then washed with 0.9% saline for the immunoassay. In the assay, standards of T_4_ were applied in triplicate, and samples in duplicate. Assay buffer (50 mM Tris, 0.9% NaCl, 0.5% BSA, 0.05% NaN_3_, 0.01% Tween 40, 20 µM diethylenetriamine-N,N,N′,N″,N″-pentaacetic acid, pH 7.75), standards of T_4_, serum samples, and europium (Eu) T_4_ BSA were added, and then incubated at room temperature for 4 hr. After three washes with 0.9% saline, europium was dissociated from the antibody-antigen complex on the surface of the wells with enhancement solution (Wallac Oy). The intensity of fluorescence from dissociated Eu was measured with a time-resolved fluorometer (1234 DELFIA fluorometer, Wallac Oy) using DOS based multicalc software. Standard curves for each assay were plotted and values were calculated automatically by the time-resolved fluorometer. The intra-assay coefficient of variation (CV) was 7.9%, and the inter- assay CV was 12.6%.

### Imprinting Procedures

The imprinting procedure was carried out according to Nevitt et al [14]. Shoji et al [19] analyzed the composition of amino acids in Toya Lake Station water, which was found to be well water containing various species of amino acids, characterized by the absence of Pro and the presence of Glu. In contrast, Toya Lake water was characterized by the presence of Pro and the absence of Glu (Table S1). Monthly variation experiments using 1400 one-year-old lacustrine sockeye salmon were conducted from March to July, 2005. The fish were divided equally into experimental and control groups. Each month, 100 fish in the experimental group were exposed to 1 µM Pro in Toya Lake Station water for 14 days from March to July. The test water was prepared by dropping 1 mM Pro into Toya Lake Station water contained in 60-L tanks until a final concentration of 1 µM Pro was reached. Fish in the control group were not exposed to Pro. After each monthly experiment, fish in the experimental and control groups were individually marked, transferred to the circular tanks, and reared until the breeding season of 2007, when the fish became sexually mature.

An experiment to determine the requirement time for imprinting was conducted in May 2006 using 2000 fish. For each exposure time, 100 fish in the experimental group were exposed to 1 µM Pro or Glu in Toya Lake Station water for 1 hour, 6 hours, 1 day, 7 days, or 14 days. The test water was prepared by dropping 1 mM Pro or Glu into Toya Station water contained in 60-L tanks until a final concentration of 1 µM was reached. Fish in the control group were not exposed to Pro or Glu. Similar to the monthly variation experiment, fish in the experimental and control group were reared until October 2007.

### Electro-Olfactogram (EOG) Recording

In order to examine whether the fish were imprinted by Pro or Glu, EOG responses to the test water were compared between the experimental fish and the control fish. The EOG response was measured according to the technique of Evans and Hara [43] in June and October from 2005 to 2007 in the monthly variation experiment, and in June and October 2006 and 2007 in the requirement time experiment. Fish were immobilized with an intramuscular injection of 3 mg/kg body weight gallamine triethodide. Gills were aerated through the mouth with an aerated solution of clove oil (0.005%), which was not allowed to contact olfactory rosettes. The responsive properties of olfactory receptor cells were recorded by using a pair of glass microelectrodes filled with 2.5% agar-saline and bridged to silver wire. With the aid of a stereomicroscope and micromanipulators, an odorant perfusion tube was inserted gently into the in-current passage of the noses, and the recording microelectrode was inserted through the ex-current passage and positioned above the midline of the rosette at the base of posterior-most lamella. A reference microelectrode was placed on the head, and a separate ground electrode was clipped to the tail of the fish. The differential electrical signal was amplified 500-fold and filtered (100-Hz low-pass) by a direct current amplifier (A–M systems, Carlsberg, Washington, USA). The signals were digitized at 10 samples per second by using Pico scope data acquisition software (Pico Technology Ltd., St. Neots, UK), and the signal amplitudes were measured in millivolts (mV). After electrode placement, the olfactory rosettes were rinsed for 30 minutes with Toya Lake Station water at a steady rate of 1 ml/min. Each odor was then pulsed for 10 seconds into Toya Lake Station water three times with a 150-second interval. EOGs were recorded in response to 0.1 mM L-serine (Ser) and the test water (1 µM Pro or Glu). The amplitude of the EOG response was quantified by the negative phasic displacement of the odor-evoked peak relative to the prestimulus electrical baseline. All of the experiments were repeated using six randomly selected test fish in each group, and the test fish were removed after the experiment. The magnitude of the response was expressed as a percentage of the response to 0.1 mM Ser dissolved in distilled water.

### Behavioral Experiment

Behavioral experiments were conducted in a two-choice test tank (Y maze) constructed at the Toya Lake Station according to the method of Shoji et al [20]. The experimental tank consisted of two upstream arms (12 m×0.6 m, 0.6 m water depth) and one pool (3 m×1.8 m, 0.6 m water depth) with an outlet at the end. During the course of the experiment, the water flow in each arm was equal at 50 L/second. Behavioral experiments were carried out from September to November of 2007 at the Toya Lake Station. Maturing and matured three-year-old male fish (average fork length, 21.0±2.0 cm; average body weight, 116.8±35.4 g) in the experimental (Pro-exposed in 2005) and control (non-exposed) groups were used in behavioral experiments. Behavioral experiments were repeated 3–4 times for each group using equal numbers of randomly selected test fish (n = 15) each time, and the test fish were removed after the experiment. Before the experiment, the fish were kept for acclimation for 3 hours in the pool, which was covered. A gate prevented the test fish from entering each arm. After the acclimation period, one of two combinations of water was simultaneously introduced to the inlet of the left and right arm for a 9 hour period: (1) Lake Toya water (control water) in both arms; and (2) the test water (1 µM Pro) in one arm and control water in the other. A highly concentrated solution of test water (2 mM Pro) was dropped into the upper reach of one arm to give a 1 µM concentration at the gate. Lake Toya water was unfamiliar to all fish in both the experimental and the control groups, and contained various species of amino acids that were different from Toya Lake Station water (Table S1 for the first supporting information table). At the beginning of the experiment, the test and control water were introduced for about 30 minutes before the gate was opened. The experiment was conducted between 7 PM and 4 AM to avoid the influence of light intensity. Fish were allowed to move freely into and out between arms. The number of fish that stayed in either arm was counted at the end of trial (4 AM). Fish that did not enter either arm at the end of trial were excluded from the analysis.

### Molecular Biological Experiment

Total RNA was extracted from the olfactory epithelium of one-year-old lacustrine sockeye salmon from February to September of 2006, by using ISOGEN (Nippongene, Toyama, Japan) according to the manufacturer's instruction. The concentration of total RNA was determined by measurement of optical density at 260/280 nm and its quantity and also integrity were verified by gel electrophoresis. A reverse transcription (RT) reaction was performed with an ExScript® RT reagent kit (TaKaRa, Shiga, Japan). Total RNA (200 ng) was used for the RT reaction in a mixture containing 1×ExScript® Buffer (50 mM KCl, 10 mM Tris–HCl, pH 8.3), 0.5 mM dNTP Mixture, 50 µM Random 6 mers, 25 U ExScript® RTase and 5 U RNase inhibitor. The reaction was performed at 42°C for 15 min, and stopped at 92°C for 2 min.

Real-time PCR was carried out with a Mx3000P QPCR System (Stratagene, La Jolla, CA, USA). The nucleotide sequences of primers and probes are shown in Table S2. The PCR reaction mixture contained 1×*Premix Ex Taq*™ (TaKaRa), 1×ROX Reference Dey ΙΙ, 100 nM each forward and reverse primers and 130 nM of fluorogenic probe. The amplification profile was 40 cycles of 95°C for 15 sec and 60°C for 1 min (annealing and extension). To determine the amount of SOIG mRNA, a full-length SOIG cDNA was used as a standard. The standard cDNA was serially diluted at the concentrations of 1×10^4^ to 1×10^10^ copies (Figure S3). In the assay, several doses of standard cDNA were applied in triplicate, and each sample cDNA prepared from total RNA was applied in duplicate. A standard sample was applied in triplicate to estimate CV within and between runs. The intra-assay CV ranged 2.2–9.8% and the inter-assay CV was 18.56%. The amount of SOIG mRNA was expressed as copies per microgram of total RNA.

### Statistical Analysis

All data are expressed as means±SEM. Statistical differences were determined using one-way analysis of variance (ANOVA) followed by Dunnett's test for EOG recording and by Tukey's test for real time PCR analysis, respectively. The behavioral selectivity of each arm in the test fish was analyzed by using a chi-square test comparing the results versus the 50∶50 expected value. Data were considered significant when *p*<0.05.

## Supporting Information

Figure S1 Changes in the condition factor (CF) of one-year-old lacustrine sockeye salmon in 2006. The bold and normal asterisks indicate significant differences in female and male, respectively. Significance was observed between June and other months in female. And significance was observed between September and April, June, July in male (p<0.05 by one-way ANOVA following Tukey's test). Values represent the means±SEM.
(0.11 MB TIF)

Figure S2 Changes in serum thyroxine (T4) levels of one-year-old lacustrine sockeye salmon in 2006. The bold and normal asterisks indicate significant differences in female and male, respectively. Significance was observed between May and other months in female. And significance was observed between February and May, June, September and May, June in male (p<0.05 by one-way ANOVA following Tukey's test). Values represent the means±SEM.
(0.12 MB TIF)

Figure S3 Typical standard curve of real-time PCR for SOIG mRNA.
(0.05 MB TIF)

Table S1 The concentration of amino acids and related substances in Toya Lake Station water and Toya Lake water
(0.06 MB TIF)

Table S2 Sequence of TaqMan probe and primers for real-time PCR analysis
(0.05 MB TIF)
