# Supplementary figures and images for: Olfactory Imprinting of Amino Acids in Lacustrine Sockeye Salmon
Source: PLoS One. 2010 Jan 8;5(1):e8633. doi: 10.1371/journal.pone.0008633 (PMC2799659; doi:10.1371/journal.pone.0008633)

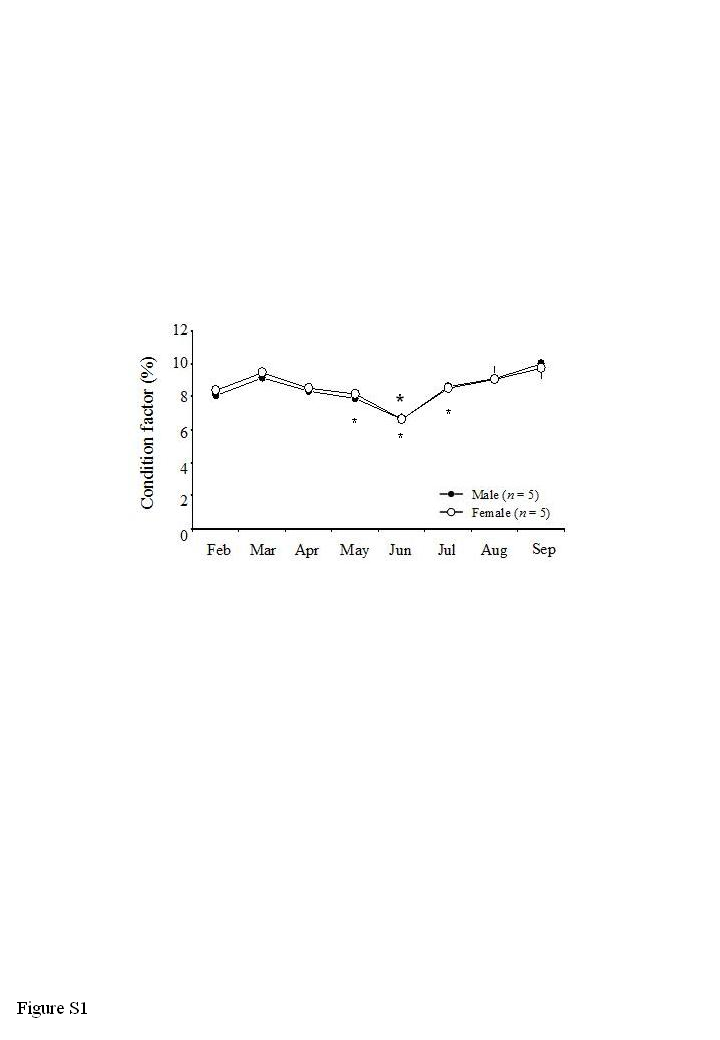

Supplement: Figure S1 — Changes in the condition factor (CF) of one-year-old lacustrine sockeye salmon in 2006. The bold and normal asterisks indicate significant differences in female and male, respectively. Significance was observed between June and other months in female. And significance was observed between September and April, June, July in male (p<0.05 by one-way ANOVA following Tukey's test). Values represent the means±SEM. (0.11 MB TIF) [file pone.0008633.s001.tif]

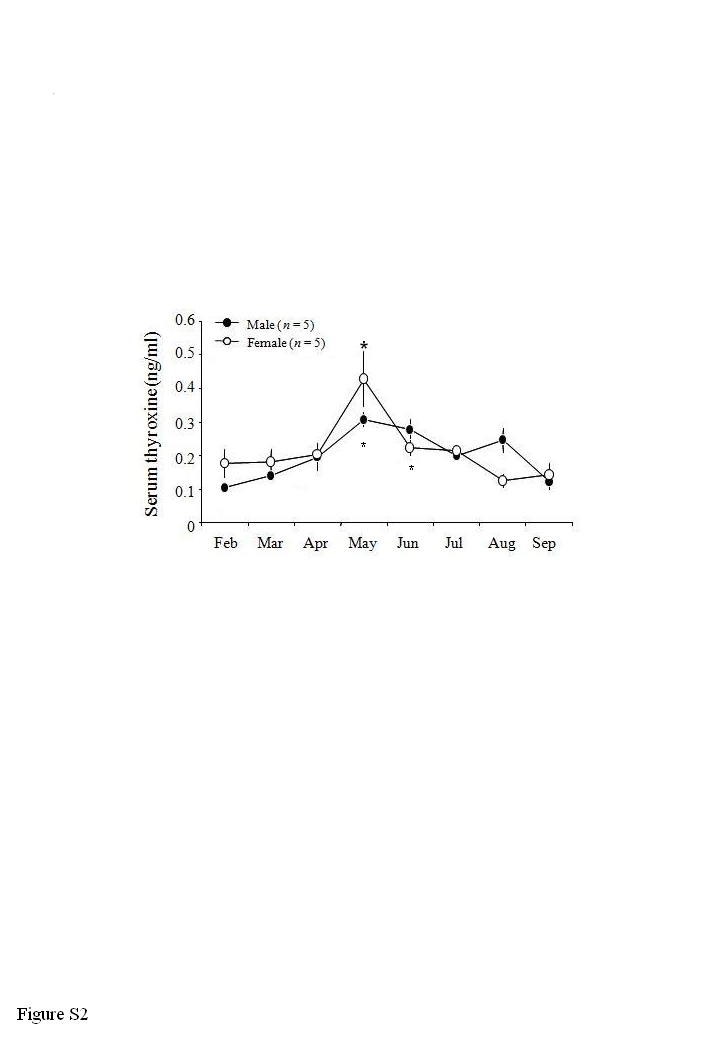

Supplement: Figure S2 — Changes in serum thyroxine (T4) levels of one-year-old lacustrine sockeye salmon in 2006. The bold and normal asterisks indicate significant differences in female and male, respectively. Significance was observed between May and other months in female. And significance was observed between February and May, June, September and May, June in male (p<0.05 by one-way ANOVA following Tukey's test). Values represent the means±SEM. (0.12 MB TIF) [file pone.0008633.s002.tif]

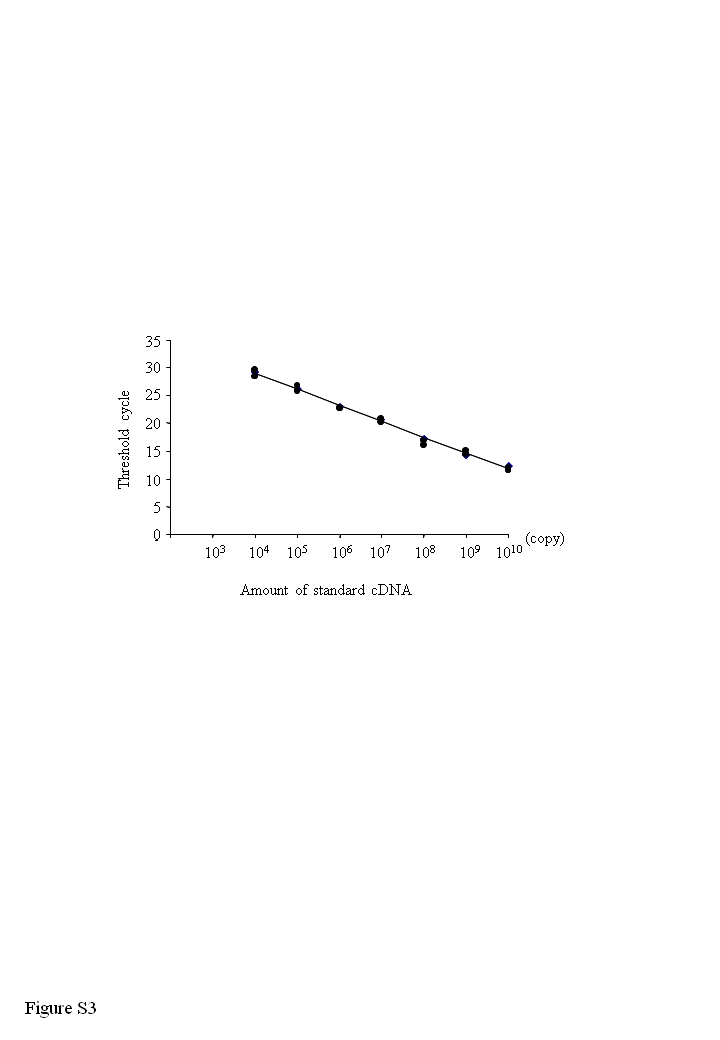

Supplement: Figure S3 — Typical standard curve of real-time PCR for SOIG mRNA. (0.05 MB TIF) [file pone.0008633.s003.tif]

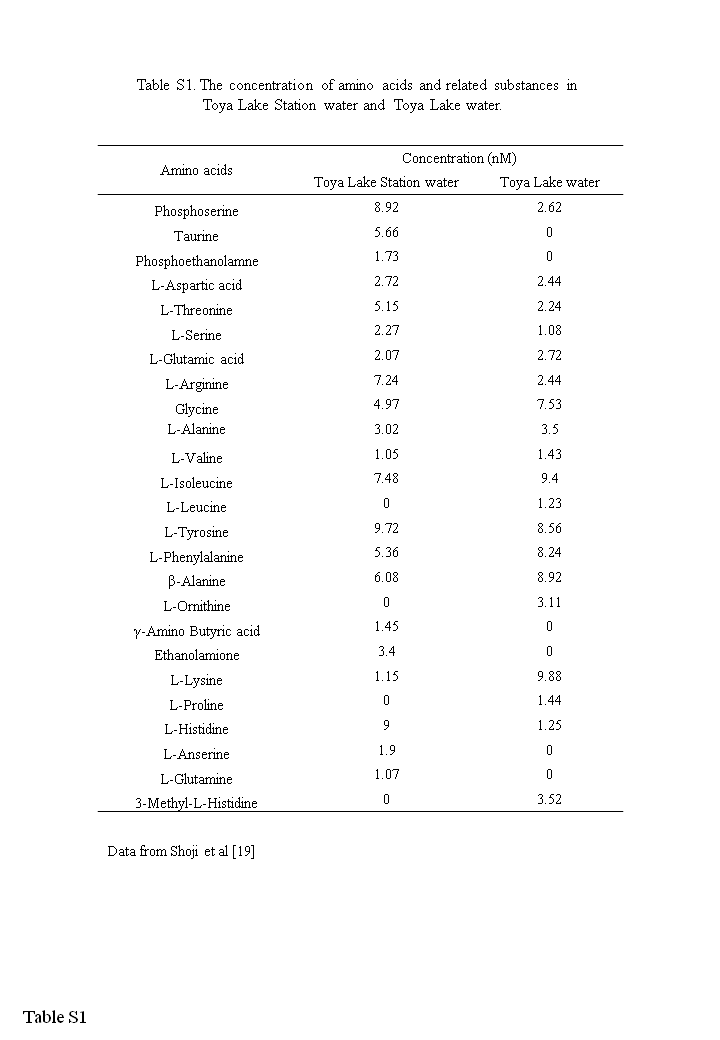

Supplement: Table S1 — The concentration of amino acids and related substances in Toya Lake Station water and Toya Lake water (0.06 MB TIF) [file pone.0008633.s004.tif]

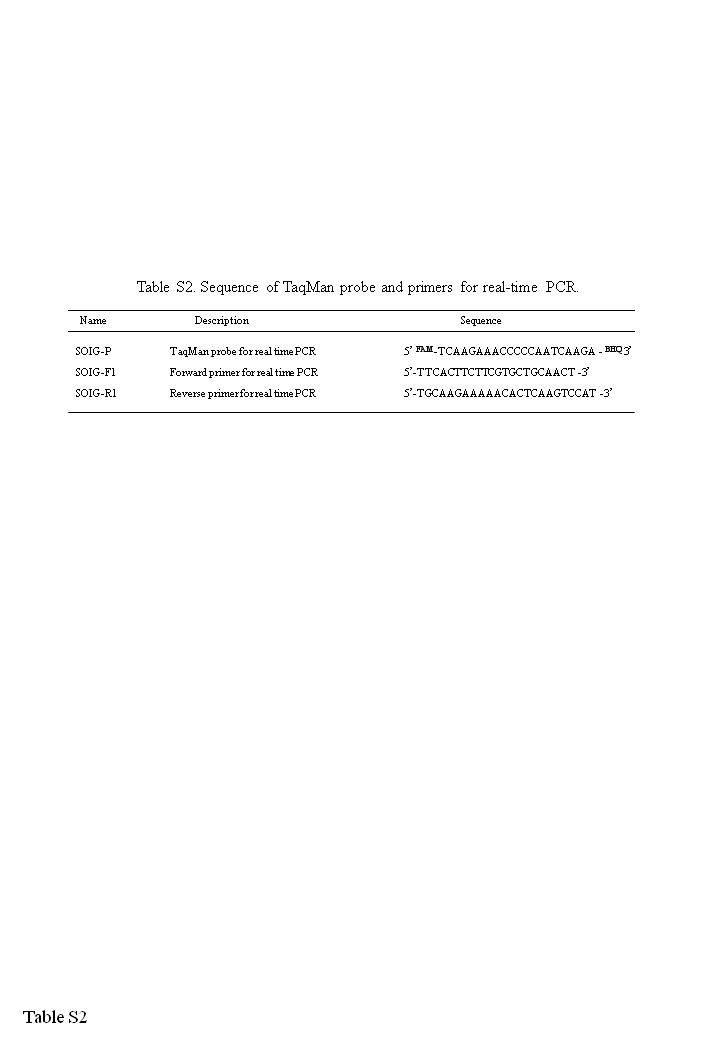

Supplement: Table S2 — Sequence of TaqMan probe and primers for real-time PCR analysis (0.05 MB TIF) [file pone.0008633.s005.tif]
